# Maternal kidney disease and composite kidney outcomes in the offspring

**DOI:** 10.1080/0886022X.2026.2745220

**Published:** 2026-10-20

**Authors:** Chien-Ning Hsu, Lung-Chih Li, Shao-Ju Chien, You-Lin Tain

**Affiliations:** ^a^ Department of Pharmacy, Kaohsiung Municipal Ta-Tung Hospital, Kaohsiung, Taiwan; ^b^ Department of Pharmacy, Kaohsiung Chang Gung Memorial Hospital, Kaohsiung, Taiwan; ^c^ School of Pharmacy, Kaohsiung Medical University, Kaohsiung, Taiwan; ^d^ Division of Nephrology, Department of Internal Medicine, Kaohsiung Chang Gung Memorial Hospital, Chang Gung University College of Medicine, Kaohsiung, Taiwan; ^e^ Institute for Translational Research in Biomedicine, Kaohsiung Chang Gung Memorial Hospital, Chang Gung University College of Medicine, Kaohsiung, Taiwan; ^f^ Division of Pediatric Cardiology, Kaohsiung Chang Gung Memorial Hospital and Chang Gung University College of Medicine, Kaohsiung, Taiwan; ^g^ Division of Pediatric Nephrology, Kaohsiung Chang Gung Memorial Hospital and Chang Gung University College of Medicine, Kaohsiung, Taiwan

**Keywords:** Maternal kidney disease, offspring, pregnancy, composite kidney outcome, chronic kidney disease, intergenerational kidney risk

## Abstract

Maternal chronic kidney disease (CKD) is associated with adverse pregnancy outcomes, but its relationship with long-term offspring kidney health remains uncertain. This retrospective maternal-child cohort study examined whether maternal CKD or reduced kidney function before or during pregnancy was associated with a composite kidney outcome in offspring. Live births in the Taiwan Mother and Child Health cohort from 2007 to 2017 were followed through 2018. In the primary cohort, maternal kidney disease was defined as claims-based CKD meeting chronicity criteria before or during pregnancy; the maternal eGFR subcohort additionally included eGFR-defined reduced kidney function. The primary outcome comprised ICD-defined CKD, reduced eGFR <60 mL/min/1.73 m^2^ or ≥30% eGFR decline at age ≥2 years, or kidney replacement therapy. Laboratory components were interpreted as reduced kidney function rather than definitive CKD unless diagnostic chronicity was documented. Models adjusted for maternal, pregnancy, offspring, and perinatal covariates. Among 79,036 children, 4,687 (5.9%) met the composite kidney outcome. Maternal kidney disease was associated with modestly higher odds of the composite outcome (aOR, 1.29; 95% CI, 1.00–1.66) and offspring all-cause mortality (aOR, 1.88; 95% CI, 1.15–3.09). Analyses of individual components of the composite outcome, sex-stratified subgroups, and trimester-specific exposure windows were exploratory, imprecise, and not adjusted for multiplicity. Maternal CKD or reduced kidney function may identify dyads who warrant long-term kidney surveillance, although causal pathways require confirmation.

## PLAIN LANGUAGE SUMMARY

Kidney disease or reduced kidney function before or during pregnancy may help identify children who could benefit from long-term kidney monitoring. In this large Taiwanese cohort, these maternal conditions were associated with modestly higher odds of clinically detected kidney outcomes and death during childhood follow-up.

## KEY MESSAGES

What is known: Maternal CKD is linked to adverse pregnancy outcomes, but evidence on long-term kidney outcomes in offspring is limited.

This study adds: Among 79,036 mother-child dyads, maternal CKD or reduced kidney function was associated with modestly higher odds of the study-specific composite kidney outcome and mortality in offspring.

Potential impact: Maternal kidney health may support risk stratification and longitudinal offspring kidney surveillance, but studies with protocolized testing, family/genetic data, and longer follow-up are needed before causal or screening recommendations can be made.

## Graphical Abstract

**Figure:**
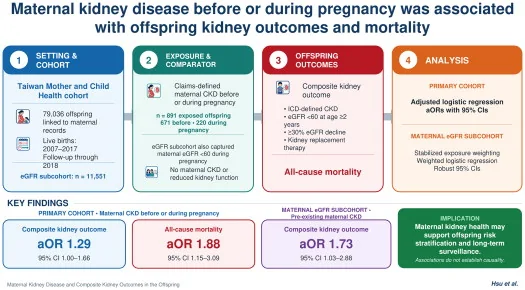

## Introduction

Chronic kidney disease (CKD) is a major global health challenge associated with lifelong morbidity, premature mortality, and substantial healthcare burden [1,2]. Maternal and perinatal conditions may shape offspring kidney health through fetal nephrogenesis, placental function, growth, and later renal reserve [3–5]. Although maternal CKD is associated with preterm birth and low birth weight, long-term offspring outcomes—including CKD, progressive kidney function loss, and kidney replacement therapy (KRT)—remain poorly characterized in large populations [6–8].

Maternal kidney disease may affect offspring kidney health through chronic vascular, metabolic, or inflammatory dysfunction; pregnancy-related renal stress; and hypertensive or placental disease [9,10]. These conditions may alter uteroplacental perfusion, nutrient delivery, fetal growth, nephron endowment, and subsequent kidney reserve in the offspring [3–7]. Medication use may also mark maternal disease severity or fetal exposure because many drugs cross the placenta and some affect fetal kidney development [11,12]. This study evaluated whether maternal claims-defined CKD or reduced kidney function before or during pregnancy was associated with a study-specific composite kidney outcome or mortality in offspring in a large Taiwanese maternal-child cohort.

## Methods

### Study population and data sources

The Mother and Child Health (MACH) study was derived from the Chang Gung Research Database (CGRD), a longitudinal electronic health record system used in kidney and maternal-child health research [13–15]. The cohort covered six Chang Gung Memorial Hospitals across Taiwan, including two medical centers and several regional and local hospitals, and accounted for approximately 10–12% of services reimbursed by Taiwan’s National Health Insurance program [16].

The MACH cohort included all live births from January 1, 2007, through December 31, 2017. Mother-infant dyads were deterministically linked using maternal identification numbers and delivery dates. The National Birth Certificate Application Database provided birth information, including sex, birth weight, and gestational age; mortality came from the National Cause of Death Statistics. Clinical records were linked to the Taiwan National Health Insurance Research Database to support follow-up from preconception through postnatal care.

The Institutional Review Board of the Chang Gung Medical Foundation, Taipei, Taiwan, approved the study (202001133B0C501) and waived informed consent because the data were retrospective and de-identified. All procedures conformed to the Declaration of Helsinki.

### Outcome and case definitions

The primary outcome was a study-specific composite kidney outcome from birth through December 31, 2018, defined by any of the following: (1) ICD-9/10-CM-defined CKD meeting chronicity criteria (≥1 inpatient diagnosis or ≥2 outpatient diagnoses at least 90 days apart) [17]; (2) eGFR <60 mL/min/1.73 m^2^ measured at age ≥2 years; (3) ≥30% eGFR decline from baseline at age ≥2 years [18]; or (4) kidney replacement therapy (KRT), including chronic dialysis or kidney transplantation, identified by procedure codes [17–19].

Laboratory components were assessed only at age ≥2 years to reduce misclassification from age-dependent physiologic variation and ensure applicability of the bedside Schwartz equation. Younger children remained eligible for ICD-defined CKD, KRT, and mortality ascertainment. When multiple serum creatinine measurements occurred on the same date, the earliest value was paired with the nearest height within 180 days. A single eGFR <60 mL/min/1.73 m^2^ could reflect transient illness, hydration, laboratory variation, or acute kidney injury and was therefore interpreted as reduced kidney function rather than definitive CKD unless supported by diagnostic chronicity or persistent abnormalities. ICD-defined CKD and KRT were ascertained regardless of age. CKD was hierarchically classified as CAKUT-related when the earliest relevant code indicated CAKUT; otherwise, it was classified as non-CAKUT. A progressive kidney event comprised KRT or ≥30% eGFR decline [18]. All-cause mortality was analyzed separately, and children without the composite outcome served as controls.

### Exposure assessment

In the primary cohort, maternal CKD required ICD-9/10-CM evidence meeting diagnostic chronicity criteria: ≥1 inpatient diagnosis or ≥2 outpatient diagnoses at least 90 days apart within 1 year [17]. Preexisting CKD was defined by qualifying evidence before conception or within the 280-day pre-pregnancy window. First qualifying evidence from conception to delivery was classified as CKD recognized during pregnancy; this category could include previously unrecognized rather than incident CKD.

In the prespecified maternal eGFR subcohort, exposure additionally included maternal eGFR <60 mL/min/1.73 m^2^ during pregnancy, calculated using the Taiwan-modified MDRD equation [20] (Supplementary Table 1). Trimester-specific categories reflected qualifying evidence within each window and could overlap when evidence occurred in more than one trimester.

An isolated maternal eGFR <60 mL/min/1.73 m^2^ during pregnancy did not establish CKD chronicity and was therefore distinguished from claims-defined CKD. Maternal acute kidney injury was excluded from the exposure framework because available data could not reliably separate it from chronic or previously unrecognized kidney disease.

### Covariates

Maternal covariates comprised age at delivery (<35 or ≥35 years), preexisting hypertension, hyperlipidemia, Charlson Comorbidity Index conditions, gestational diabetes, gestational hypertension, preeclampsia, anemia, other pregnancy complications, and medication use before and during pregnancy [21–25]. Medication variables included nonsteroidal anti-inflammatory drugs (NSAIDs), aspirin, systemic glucocorticoids, antimicrobial agents, and aminoglycosides (Supplementary Table 1). To avoid redundant adjustment, aminoglycosides were described as an antimicrobial subclass but were not entered with overall antimicrobial use. Covariates supported confounding control and description; individual coefficients were not interpreted causally, and medication associations could reflect indication or disease severity because dose, adherence, and exact timing were unavailable. Separate estimates in Table 4 were descriptive PAF inputs.

Measured offspring and design covariates included birth period (2007–2009, 2010–2012, 2013–2015, or 2016–2017), sex, plurality (singleton, twins, or triplets), and congenital anomalies excluding CAKUT. Potential perinatal mediators included gestational age (<27, 27 to <34, 34 to <37, or ≥37 weeks), birth weight (<2.5, 2.5 to <4, or ≥4 kg), and fetal growth classification (small, appropriate, or large for gestational age). Gestational age was modeled in clinically recognized strata to accommodate nonlinearity. Neonatal acute kidney injury could not be reliably ascertained because laboratory definitions and inpatient coding were not uniformly available.

### Statistical analysis

Baseline characteristics were summarized as frequencies and percentages or as means with standard deviations or medians with interquartile ranges. Associations between maternal kidney disease or reduced kidney function and the offspring composite outcome were estimated by multivariable logistic regression and reported as adjusted odds ratios (aORs) with 95% confidence intervals (CIs). Birth period was included to partially account for differences in observation duration and eligibility for laboratory outcome ascertainment. Sex-stratified analyses were exploratory and were not interpreted as formal tests of effect modification.

Population-attributable fractions (PAFs) were calculated using Levin’s formula to compare the relative population burden associated with maternal factors [26].

A conceptual directed acyclic graph (DAG) formalized assumptions about confounding, mediation, and ascertainment (Supplementary Figure 1). Measured adjustment covariates were grouped as maternal/pregnancy factors (maternal age, preexisting comorbidities, pregnancy complications, and medication use) and offspring/design factors (offspring sex, plurality, birth period, and congenital anomalies excluding CAKUT). Gestational age, birth weight, and fetal growth classification were considered potential perinatal mediators because they may lie downstream of maternal kidney disease. Accordingly, models including these variables were interpreted as perinatal-adjusted associations rather than estimates of the total causal effect [27,28]. The DAG also represented unmeasured familial/shared factors and healthcare-contact/testing pathways that may contribute to residual confounding and differential ascertainment.

Trimester-specific windows were evaluated in the maternal eGFR subcohort using stabilized inverse probability-of-exposure weights followed by weighted logistic regression with additional offspring and perinatal adjustment [29]. Propensity scores included maternal age, preexisting comorbidities, medication use, and pregnancy complications (Supplementary Table 2); robust sandwich estimators generated 95% CIs. A component of composite outcomes, sex-stratified, and trimester-specific analyses were prespecified but exploratory and were not adjusted for multiplicity. A maternal kidney disease-by-gestational-age interaction was not tested because gestational age may be a mediator and exposed outcome events were sparse. All tests were two-sided, with *p* < 0.05 considered statistically significant. Analyses used SAS version 9.4 (SAS Institute, Cary, NC, USA).

## Results

### Cohort and offspring characteristics

Among 79,036 children, 4,687 (5.9%) met the study-specific composite kidney outcome (Figure 1), including 3,517 (4.45%) with ICD-defined CKD and 893 (1.13%) with reduced eGFR after age 2 years. Children with the composite outcome were more often born in 2016–2017 (18.80% vs. 13.90%); this distribution was not interpreted as a temporal trend. Low birth weight (18.0% vs. 16.7%) and large-for-gestational-age birth (20.3% vs. 18.2%) were also slightly more common (Table 1).

**Figure 1.**
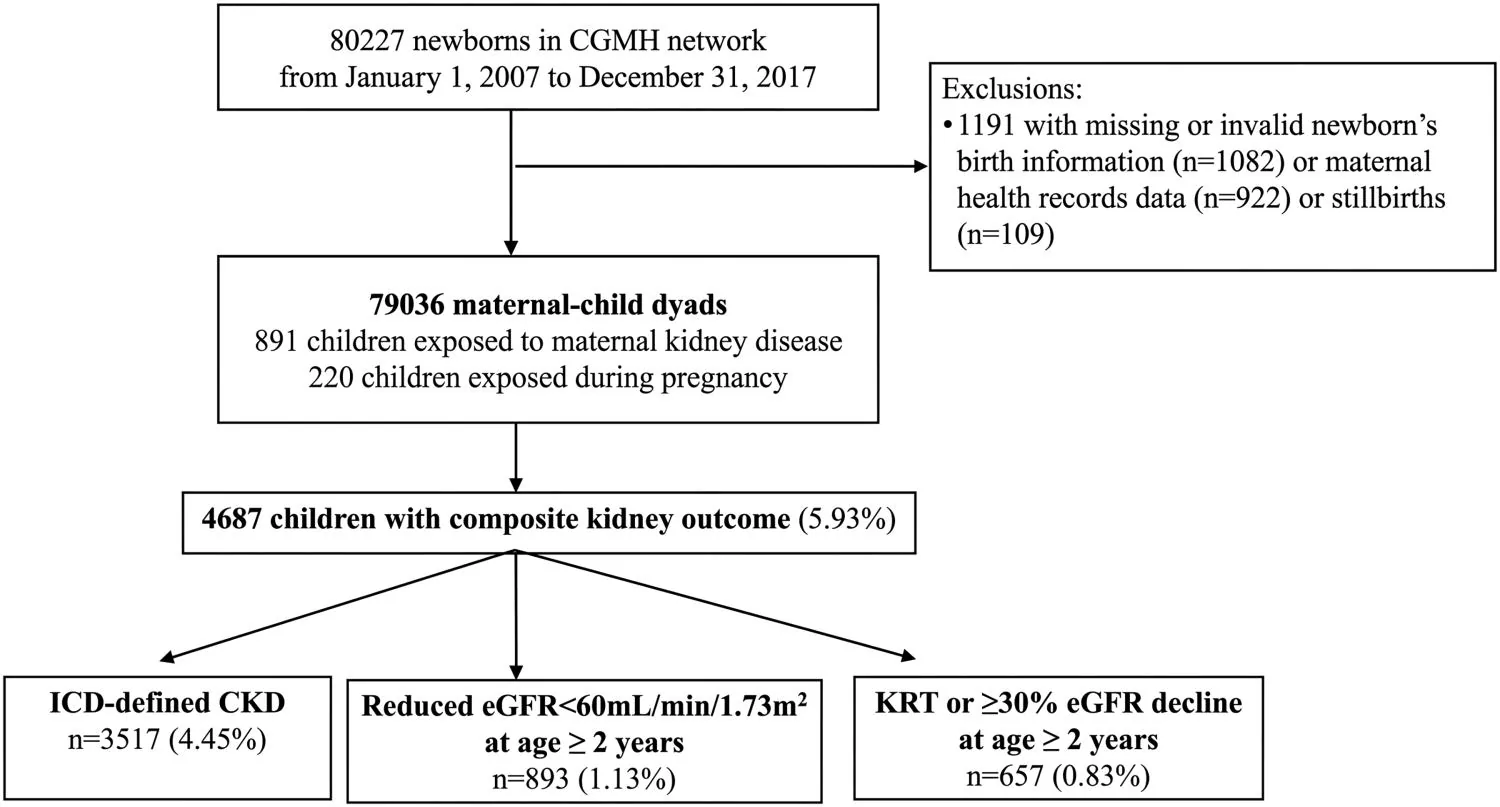
Data sources and linkage of the Mother and Child Health (MACH) cohort. The composite kidney outcome included ICD-defined CKD, reduced eGFR <60 mL/min/1.73 m^2^ at age ≥2 years, ≥30% eGFR decline at age ≥2 years, or KRT. A single reduced eGFR value was interpreted as reduced kidney function rather than definitive CKD without diagnostic chronicity. Components could overlap. Exclusion subcategories for missing or invalid birth information and maternal records were also not mutually exclusive. CGMH, Chang Gung Memorial Hospital; CKD, chronic kidney disease; eGFR, estimated glomerular filtration rate; KRT, kidney replacement therapy.

**Table 1.** Offspring characteristics by composite kidney outcome status in the primary cohort.

Offspring and perinatal characteristics	All children (*n* = 79,036)	Composite outcome (*n* = 4,687)	No composite outcome (*n* = 74,349)	*p* value
Birth period, n (%)				<0.0001
2007–2009	24,444	1,408 (30.04)	23,036 (30.98)	
2010–2012	22,177	1,287 (27.46)	20,890 (28.10)	
2013–2015	21,196	1,111 (23.70)	20,085 (27.01)	
2016–2017	11,219	881 (18.80)	10,338 (13.90)	
Sex, n (%)				<0.0001
Male	41,427	2,747 (58.61)	38,680 (52.02)	
Female	37,609	1,940 (41.39)	35,669 (47.98)	
Birth weight, kg, n (%)				0.0357
<2.5	13,281	845 (18.03)	12,436 (16.73)	
2.5 to <4	64,304	3,747 (79.94)	60,557 (81.45)	
≥4	1,451	95 (2.03)	1,356 (1.82)	
Gestational age, weeks, n (%)				0.022
<27	770	51 (1.09)	719 (0.97)	
27 to <34	4,708	266 (5.68)	4,442 (5.97)	
34 to <37	9,255	611 (13.04)	8,644 (11.63)	
≥37	64,303	3,759 (80.20)	60,544 (81.43)	
Fetal growth classification, n (%)				0.0007
SGA	8,796	543 (11.59)	8,253 (11.10)	
AGA	55,735	3,195 (68.17)	52,540 (70.67)	
LGA	14,505	949 (20.25)	13,556 (18.23)	
Plurality, n (%)				0.2556
Singleton	73,257	4,363 (93.09)	68,894 (92.66)	
Twins	5,560	316 (6.74)	5,244 (7.05)	
Triplets	219	8 (0.17)	211 (0.28)	

Composite kidney outcome: study-specific composite of ICD-defined CKD, reduced eGFR, ≥30% eGFR decline, or KRT. SGA, small for gestational age; AGA, appropriate for gestational age; LGA, large for gestational age.

P values compare children with and without the composite kidney outcome; values for multicategory variables are from overall chi-square tests.

### Maternal characteristics by offspring composite kidney outcome status

During pregnancy, NSAID or aspirin use (41.7% vs. 39.6%; *p* = 0.0037) and antimicrobial use (56.2% vs. 54.1%; *p* = 0.0044) were modestly more common among mothers of children with the composite outcome. Before pregnancy, NSAID or aspirin, glucocorticoid, antimicrobial, and aminoglycoside use showed similar modest differences (Table 2).

**Table 2.** Maternal characteristics by offspring composite kidney outcome status.

Maternal covariates	All children (*n* = 79,036)	Composite outcome (*n* = 4,687)	No composite outcome (*n* = 74,349)	*p* value
Maternal age, n (%)				0.2293
<35 years	58,548	3,507 (74.82)	55,041 (74.03)	
≥35 years	20,488	1,180 (25.18)	19,308 (25.97)	
Medication use during pregnancy, n (%)				
NSAID/aspirin	31,358	1,954 (41.69)	29,404 (39.55)	0.0037
Glucocorticoid	5,328	346 (7.38)	4,982 (6.70)	0.0712
Antimicrobial	42,846	2,635 (56.22)	40,211 (54.08)	0.0044
Aminoglycoside	4,238	273 (5.82)	3,965 (5.33)	0.1473
Medication use before pregnancy, n (%)				
NSAID/aspirin	61,614	3,748 (79.97)	57,866 (77.83)	0.0006
Glucocorticoid	29,809	1,908 (40.71)	27,901 (37.53)	<0.001
Antimicrobial	57,408	3,518 (75.06)	53,890 (72.48)	0.0001
Aminoglycoside	3,481	247 (5.27)	3,234 (4.35)	0.0029
Pregnancy complications, n (%)				
Gestational diabetes	1,273	61 (1.30)	1,212 (1.63)	0.083
Gestational hypertension	859	66 (1.41)	793 (1.07)	0.0287
Preeclampsia	485	32 (0.68)	453 (0.61)	0.5323
Anemia	40	3 (0.06)	37 (0.05)	0.642
Other complications	51,737	3,202 (68.32)	48,535 (65.28)	<0.001
Preexisting comorbidities, n (%)				
Hypertension	1,578	114 (2.43)	1,464 (1.97)	0.0279
Hyperlipidemia	2,612	157 (3.35)	2,455 (3.30)	0.8594
Maternal kidney disease	891	72 (1.54)	819 (1.10)	0.0063

Composite kidney outcome: study-specific composite of ICD-defined CKD, reduced eGFR <60 mL/min/1.73 m^2^, ≥30% eGFR decline, or KRT.

Maternal kidney disease in the primary cohort was defined solely by ICD-defined CKD and was identified among mothers of 891 children: 671 before pregnancy and 220 first recognized during pregnancy. Other pregnancy complications comprised conditions listed in Supplementary Table 1 after excluding gestational diabetes, gestational hypertension, preeclampsia, anemia, and maternal kidney disease.

Gestational hypertension was more frequent among mothers of children with the composite outcome (1.4% vs. 1.1%; *p* = 0.0287), whereas gestational diabetes and preeclampsia did not differ significantly. Other pregnancy complications (excluded gestational hypertension, hyperlipidemia, diabetes, preeclampsia and anemia) were more common in the composite outcome group (68.3% vs. 65.3%; *p* < 0.001). Maternal CKD was identified among mothers of 891 children: 671 before pregnancy and 220 first recognized during pregnancy (Table 2).

### Association between maternal kidney disease and offspring outcomes

The composite kidney outcome occurred in 8.08% of offspring exposed to maternal CKD and 5.91% of unexposed offspring (Table 3). After multivariable adjustment, maternal CKD was associated with modestly higher odds of the offspring composite kidney outcome (aOR, 1.29; 95% CI, 1.00–1.66) (Figure 2). The estimate for CAKUT-related CKD was elevated (aOR, 1.42; 95% CI, 1.02–1.97), whereas estimates for non-CAKUT CKD (aOR, 1.19; 95% CI, 0.75–1.90) and progressive kidney outcomes (aOR, 1.41; 95% CI, 0.75–2.65) were imprecise and did not establish phenotype-specific differences. Maternal CKD was also associated with offspring all-cause mortality (aOR, 1.88; 95% CI, 1.15–3.09).

**Figure 2.**
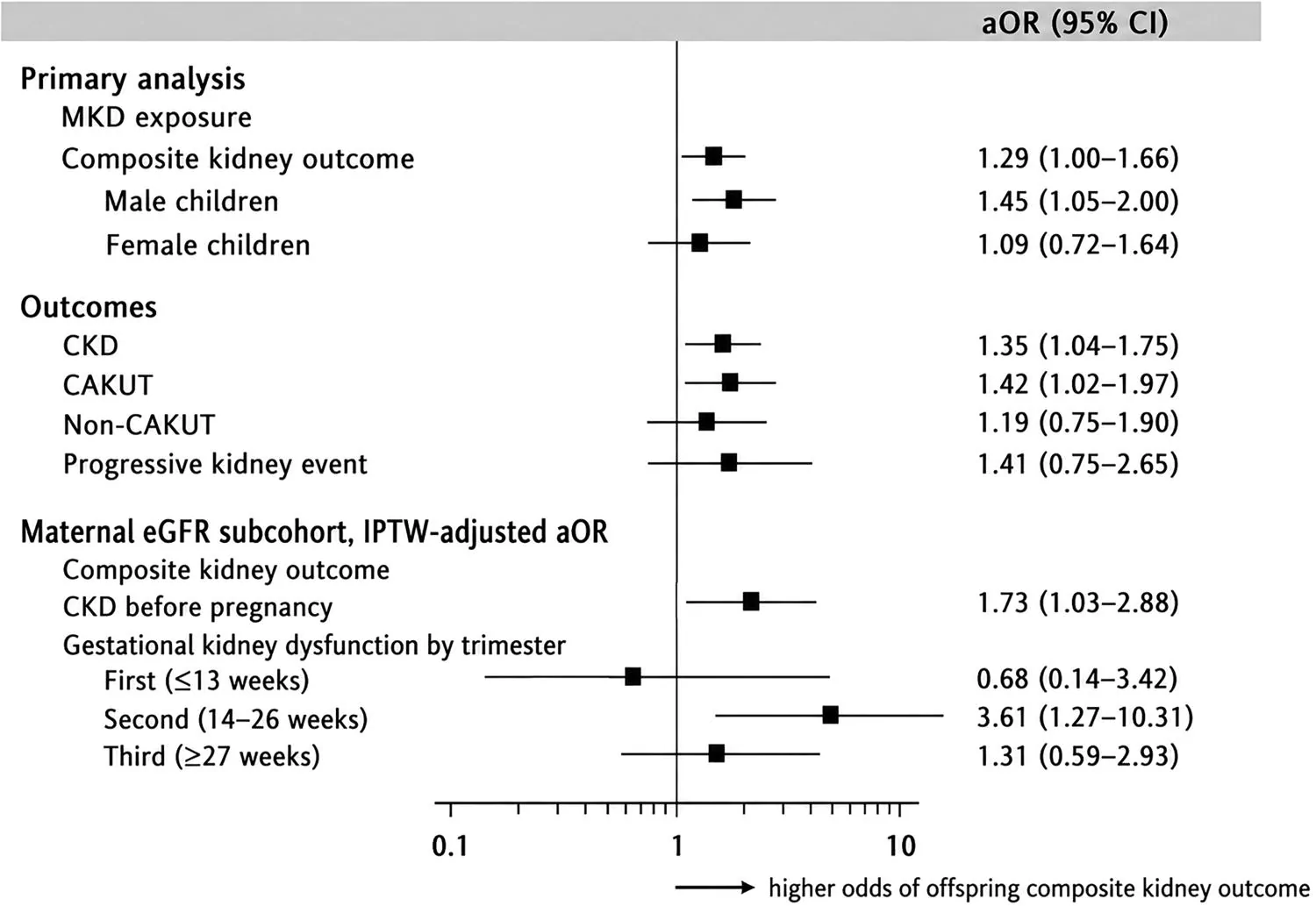
Associations between maternal kidney disease or reduced kidney function and observed offspring composite kidney outcomes. In the primary cohort, adjusted odds ratios (aORs) and 95% confidence intervals (CIs) were estimated by multivariable logistic regression including maternal and pregnancy covariates (age, medication use before and during pregnancy, preexisting conditions, and pregnancy complications), offspring/design covariates (birth period, sex, plurality, and congenital anomalies excluding CAKUT), and potential perinatal mediators (gestational age, birth weight, and fetal growth classification). In the maternal eGFR subcohort (n = 11,551), stabilized inverse probability-of-exposure weighting balanced measured maternal covariates, followed by weighted logistic regression with offspring and perinatal adjustment. Robust sandwich estimators generated 95% CIs.

**Table 3.** Offspring composite kidney outcomes and components by maternal kidney disease exposure status.

Offspring outcomes	All children (*n* = 79,036)	Maternal kidney disease (*n* = 891)	No maternal kidney disease (*n* = 78,145)
Composite kidney outcome, n (%)	4,687 (5.93)	72 (8.08)	4,615 (5.91)
CKD	4,302 (5.44)	68 (7.63)	4,234 (5.42)
ICD-defined CKD	3,517 (4.45)	55 (6.17)	3,462 (4.43)
CAKUT-related CKD	2,408 (3.05)	42 (4.71)	2,366 (3.03)
Non-CAKUT CKD	1,459 (1.85)	20 (2.24)	1,439 (1.84)
Reduced eGFR <60 mL/min/1.73 m^2^ at age ≥2 years	893 (1.13)	15 (1.68)	878 (1.12)
Progressive kidney outcome	657 (0.83)	11 (1.23)	646 (0.83)
Kidney replacement therapy	40 (0.05)	<5	—
≥30% eGFR decline at age ≥2 years	623 (0.79)	9 (1.01)	614 (0.79)
All-cause mortality, n (%)	777 (0.98)	24 (2.69)	753 (0.96)

CKD, chronic kidney disease; eGFR, estimated glomerular filtration rate; KRT, kidney replacement therapy.

Composite kidney outcome: study-specific composite of ICD-defined CKD, reduced eGFR <60 mL/min/1.73 m^2^, ≥30% eGFR decline, or KRT. Maternal kidney disease in the primary cohort was defined solely by ICD-defined CKD. KRT cells with counts <5 were suppressed under Taiwan NHIRD privacy regulations.

### Sex-stratified and maternal eGFR subcohort analyses

The estimate was higher among male offspring (aOR, 1.45; 95% CI, 1.05–2.00) than among female offspring (aOR, 1.09; 95% CI, 0.72–1.64), but the CIs overlapped and did not establish a sex-specific difference (Figure 2).

In the maternal eGFR subcohort of 11,551 mother-offspring pairs, 328 children (2.84%) were exposed to maternal ICD-defined CKD or eGFR <60 mL/min/1.73 m^2^. Preexisting CKD was more frequent among children with the composite outcome than among controls (2.61% vs. 1.57%; *p* = 0.0329), whereas CKD recognized during pregnancy did not differ significantly (1.78% vs. 1.45%; *p* = 0.4705). Maternal eGFR <60 mL/min/1.73 m^2^ during pregnancy was also more frequent among cases (5.01% vs. 2.43%), although timing-specific comparisons involved few exposed events (Supplementary Tables 2 and 3).

In weighted analyses, preexisting maternal CKD was associated with higher odds of the offspring composite kidney outcome (aOR, 1.73; 95% CI, 1.03–2.88). Reduced maternal kidney function identified in the second trimester had the highest point estimate (aOR, 3.61; 95% CI, 1.27–10.31), based on 49 exposed mother-child pairs and 8 cases; first- and third-trimester estimates were imprecise and did not show the same pattern. Sparse events precluded conclusions about a sensitive gestational window or gestational-age effect modification.

### Population-attributable fraction

The PAF for maternal kidney disease was 0.33% (Table 4), consistent with low exposure prevalence despite elevated individual-level odds. Other pregnancy complications had the largest individual PAF (6.14%), and the evaluated maternal factors yielded a combined modeled PAF of 18.36%. These estimates describe relative population burden and should not be interpreted as causal or preventable fractions.

**Table 4.** Associations between maternal factors and the offspring composite kidney outcome and population-attributable fractions.

Maternal health condition	Number exposed	Exposure prevalence (%)	aOR	PAF (%)
ICD-defined maternal CKD	891	1.13	1.29	0.33
Medication use during pregnancy				
NSAID/aspirin	31,358	39.68	1.04	1.56
Glucocorticoid	5,328	6.74	1.02	0.13
Antimicrobial	42,846	54.21	1.03	1.60
Medication use before pregnancy				
NSAID/aspirin	61,614	77.96	1.04	3.02
Glucocorticoid	29,809	37.72	1.06	2.21
Antimicrobial	57,408	72.64	1.05	3.50
Pregnancy complications				
Gestational diabetes	1,273	1.61	0.68	−0.52
Gestational hypertension	859	1.09	1.17	0.18
Preeclampsia	485	0.61	0.90	−0.06
Other complications	51,737	65.46	1.10	6.14
Preexisting comorbidities				
Hypertension	1,578	2.00	1.14	0.28
Hyperlipidemia	2,612	3.30	0.91	−0.30

PAF was calculated using Levin’s equation and the prevalence of exposure in the population (Pe) [25].

The adjusted odds ratio (aOR) was used to estimate the risk ratio, as the offspring composite kidney outcome incidence was <10%. PAF estimates for individual maternal factors should be interpreted descriptively and not as evidence of causal effects. Other pregnancy complications comprised pregnancy-related conditions listed in Supplementary Table 1 after excluding gestational diabetes, gestational hypertension, preeclampsia, anemia, and maternal kidney disease.

## Discussion

### Principal findings

Maternal CKD or reduced kidney function before or during pregnancy was associated with modestly higher odds of a study-specific composite kidney outcome and all-cause mortality in offspring. The findings extend evidence beyond adverse birth outcomes but require validation in independent cohorts.

### Comparison with prior studies

Previous studies have focused mainly on preterm delivery, low birth weight, small-for-gestational-age birth, congenital malformations, and CAKUT [3,6,8,23,24,30,31]. By evaluating ICD-defined CKD, reduced eGFR, eGFR decline, KRT, and mortality during follow-up, the present study extends evidence to postnatal kidney morbidity beyond birth outcomes and structural diagnoses.

The exposure was intentionally broad: the primary cohort captured claims-defined maternal CKD meeting chronicity criteria, whereas the eGFR subcohort additionally captured reduced kidney function recognized during pregnancy. The association therefore reflects the prognostic relevance of maternal kidney health across preconception and pregnancy, not distinct biological effects of established CKD versus pregnancy-related dysfunction or differences in ascertainment between exposure definitions.

### Biological interpretation

The mechanisms underlying the association cannot be separated. Potential explanations include altered uteroplacental perfusion, impaired fetal growth or nephron endowment, inherited susceptibility, and differential clinical ascertainment [9,32]. Although nephrogenesis spans gestation [6], the trimester-specific data were too sparse to identify a sensitive window.

The DAG identified gestational age, birth weight, and fetal growth classification as potential perinatal mediators. Models including these variables estimate perinatal-adjusted associations and do not separate direct, indirect, or total effects.

### Medication use and health status during pregnancy

Medication variables were covariates and may mark treatment indication or maternal disease severity, as well as fetal exposure because many drugs cross the placenta [11,22]. The medication classes examined − NSAIDs, aspirin, systemic glucocorticoids, antimicrobial agents, and aminoglycosides − have heterogeneous indications and should not be interpreted as representing a single pathway or causal drug effects.

Inverse point estimates for gestational diabetes (aOR, 0.68) and preexisting hyperlipidemia (aOR, 0.91) in Table 4 should not be interpreted as protective. Residual confounding, clinical monitoring or treatment differences, exposure misclassification, multiple exploratory comparisons, and chance remain plausible explanations.

### CAKUT, familial susceptibility, and genetic interpretation

Shared genetic and familial susceptibility remains an important source of unmeasured heterogeneity. CAKUT is etiologically diverse; monogenic variants and pathogenic copy-number variants explain some cases, whereas sporadic, environmental, epigenetic, developmental, and gene-environment mechanisms contribute to others [33,34]. Because paternal kidney disease or CAKUT, parental genotype, and detailed family history were unavailable, maternal intrauterine effects could not be separated from inherited or shared susceptibility. The imprecise non-CAKUT estimate and few exposed cases also precluded conclusions about differential associations by CKD phenotype.

### Strengths and limitations

Strengths include the large multicenter maternal-child cohort, linkage of diagnostic and laboratory data, follow-up within an integrated health system, and evaluation of kidney outcomes beyond birth outcomes and congenital anomalies. The maternal eGFR subcohort provided laboratory exposure data and exploratory timing analyses, but was not population-representative.

Several limitations warrant consideration. First, maternal exposure ascertainment was selective. The eGFR subcohort represented 14.6% of the primary cohort and likely included mothers with greater disease severity, more healthcare contact, or specific testing indications, creating potential surveillance bias. Conservative claims criteria may also have missed mild, subclinical, proteinuric, or untested CKD. The Taiwan-modified MDRD equation is locally calibrated but not validated for pregnancy; physiologic hyperfiltration and trimester-related creatinine changes may cause misclassification [7,35]. Maternal eGFR <60 mL/min/1.73 m^2^ was therefore interpreted as reduced kidney function rather than definitive CKD without diagnostic chronicity.

Second, offspring outcomes depended on clinical encounters and testing, potentially missing subclinical disease while preferentially detecting children with greater healthcare contact. A single eGFR <60 mL/min/1.73 m^2^ at age ≥2 years could reflect transient illness, hydration, laboratory variation, or acute kidney injury and was interpreted as reduced kidney function rather than CKD in isolation. The composite outcome should therefore be viewed as a clinically detected kidney signal rather than uniformly confirmed CKD.

Third, follow-up duration and coding era varied by birth period. Children born in 2016–2017 had the shortest follow-up but were overrepresented among those with the composite outcome, potentially reflecting testing patterns or the ICD-9-CM to ICD-10-CM transition rather than a temporal increase in kidney disease. Birth-period adjustment partly addressed these differences, and no single-coding-era sensitivity analysis was available.

Gestational age, birth weight, and fetal growth classification were included together despite the derivation of fetal growth classification from the other two measures. Formal collinearity diagnostics and nonredundant reparameterized models were unavailable; unstable individual perinatal coefficients and imprecision in the maternal CKD estimate cannot be excluded.

Finally, proteinuria and albuminuria were unavailable, as were maternal and neonatal acute kidney injury, paternal kidney disease, sibling history, detailed family history, and genetic testing. These gaps limited CKD phenotyping and control of familial or genetic confounding. Findings from this integrated Taiwanese health system may inform comparable maternal and pediatric settings, but validation in more diverse populations is needed.

## Conclusion

Maternal CKD or reduced kidney function before or during pregnancy was associated with modestly higher odds of a study-specific composite kidney outcome and mortality in offspring. Maternal kidney health may support risk stratification and longitudinal surveillance, but ascertainment, potential mediation, and sparse subgroup data preclude causal inference. Longer follow-up, protocolized kidney testing, family and genetic information, and more precise maternal and early-life phenotyping are needed.

## Supplementary Material

R2_SUPL_RenalFailure_MKD_260821clean copy.docx

## Data Availability

Individual-level data are not publicly available because of legal, ethical, and governance restrictions protecting patient privacy and use of the CGRD and linked national registries. Aggregate data are provided in the Article and Supplementary Material. Further details may be available from the corresponding author on reasonable request, subject to institutional approval and applicable data-use agreements.
