# Supplementary material for: Maternal kidney disease and composite kidney outcomes in the offspring
Source: Ren Fail. 2026 Oct 20;48(1):2745220. doi: 10.1080/0886022X.2026.2745220 (PMC13689789; doi:10.1080/0886022X.2026.2745220)
Supplement: Supplementary file 1 [file IRNF_A_2745220_SM3651.docx]

Maternal Kidney Disease and Composite Kidney Outcomes in the Offspring

**Supplementary Material**

Supplementary Table 1. Diagnostic and medication codes used to define maternal health conditions, exposures, and offspring outcomes

Supplementary Table 2. Offspring kidney outcomes by maternal kidney disease status and timing in the maternal eGFR subcohort (n=11,551).

Supplementary Table 3. Maternal characteristics by offspring composite kidney outcome status in the maternal eGFR subcohort (n=11,551).

Supplementary Figure 1. Conceptual directed acyclic graph for the association between maternal kidney disease and observed offspring composite kidney outcomes.

Supplementary Table 1. Diagnostic and Medication Codes Used to Define Maternal Health Conditions, Exposures, and Offspring Outcomes

| **Offspring outcomes e** | | **Code** | **Timing and Definition** |
| --- | --- | --- | --- |
| **Chronic kidney disease diagnoses in childhood [1, 2]** | | | ≥1 ICD-9/10 codes in inpatient setting or ≥2 ICD-9/10 codes in outpatient setting apart ≥90 days and <365 days, whichever came latest was defined as the date of CKD in post-natal follow-up   Kidney replacement therapy was based on the procedure codes and billing codes for chronic dialysis (3 continuous months and 1 time in each month) and kidney transplantation. |
| Congenital anomalies of kidney and urinary tract (CAKUT) | | **Renal agenesis, hypoplasia, or dysplasia**  ICD-9: 589, 7530, 75315; ICD-10: N270, N271, N279, Q600, Q601, Q602, Q603, Q604, Q605, Q606, Q614 |  |
|  |  | **Cystic kidney disease**  ICD-9:7531, 75310, 75311, 75312, 75313, 75314, 75316, 75319; ICD-10: Q6100, Q6101, Q6102, Q6111, Q6119, Q612, Q613, Q615, Q618, Q619 |  |
|  |  | **Other anomalies of kidney**  ICD-9: 753, 7533 (e.g., accessory kidney, giant kidney, ectopic kidney)  ICD-10: Q630, Q631, Q632, Q633, Q638, Q639 |  |
|  |  | U**rinary tract dilation and anomalies of the urethra or ureters (UTD)**  ICD-9: 591, 596, 7532, 75320, 75321, 75322, 75329, 7534, 7535, 7536,7537,7538;  ICD-10: N131, N1330, N1339, N320, Q620, Q6210, Q6211, Q6212, Q6231, Q6232, Q6239, Q624, Q625, Q6260, Q6261, Q6262, Q6263, Q6269, Q627, Q628,  Q6410, Q6411, Q6412, Q6419, Q642,  Q6431, Q6432, Q6433, Q6439,  Q644, Q645, Q646, Q647, Q648, Q649 |  |
|  |  | **Vesicoureteral-reflux with reflux nephropathy (VUR)**  ICD-9: 59371, 59372  ICD-10:N13721, N13722, N13731, N13732 |  |
| Non-CAKUT | | **Glomerular disease:**  ICD-9: 580, 581,582,583,  ICD-10: N00, N01, N03, N04, N05, N08, N14, N159 (5838/5839)  ***Systemic Lupus Erythematosus (SLE)***  ICD-9: 7100; ICD-10: M320, M3210, M3219, M328, M329  **Proteinuria**  ICD: 7910, ICD: N02 (not included: R310, R319, R311,R312)  **Other glomerular disease**  Diabetes with renal manifestations:  ICD-9: 2504, ICD-10: E1021, E1022, E1029, E1121, E1122, E1129, E1165  Renal artery aneurysm: ICD-9: 4421, ICD-10: I722  Goodpasture’s syndrome/ Wegener granulomatosis:  ICD-9: 44621,4464, ICD-10: M310, M3119, M3130  Hemolytic-uremic syndrome: ICD-9: 28311, ICD-10:D593  Henoch-Schonlein purpura: ICD-9: 2870, ICD-10: N07  Rheumatic arthritis: ICD-9: 714, ICD-10:M05 |  |
|  |  | **Chronic kidney diseases**  ICD-9: 585, 586, V451  ICD-10: N18, N19, Z992 |  |
|  |  | **Other non-glomerular disease**  Hyperaldosteronism:  ICD-9: 2551, ICD-10:'E2601' 'E2602' 'E2609' 'E261' 'E2681' 'E2689' 'E269'  Atrophy of kidney: ICD-9: 587, ICD-10: N261, N269  Renal osteodystrophy: ICD-9: 588, 5881, 5888, 5889, ICD-10: N25 N250, N251, N2581, N2589, N259  Stone: ICD-9: 592, ICD-10: N200,  Hyperplasia of renal artery: ICD-9**:**4473, ICD-10: I7773  Bladder:  ICD-9: 59654, ICD-10: N310, N311, N319  Urinary obstruction: ICD9: 5996; ICD-10: N139 |  |
|  |  | **Hypertensive, lipidoses, gout, liver related chronic kidney diseases**  Hypertension:  ICD-9: 4031, 40492, 40493, 40412, 40413; ICD-10: I129, I120, I1311, I132; |  |
| Congenital anomalies by system | | | |
|  | Nerves | ICD-9: 740-742  ICD-10: Q000, Q001, Q002, Q050-Q059, Q0700-Q0703, Q01-Q04, Q06, Q07 | ≥1 ICD-9/10 codes in out- or inpatient setting |
|  | Ear/Nose/Throat | ICD-9: 743, 744, 748.1, 749  ICD-10: Q100-Q107, Q110-Q113, Q120-Q124, Q128, Q129, Q130-Q135, Q1381, Q1389, Q139, Q140-Q143, Q148-Q150, Q158, Q159, Q16-Q18, Q30.1, Q30.2, Q30.8, Q35-Q37, Q385 |  |
|  | Heart or Circulatory | ICD-9: 745, 746, 747; ICD-10: Q20-Q24, Q25-Q28 |  |
|  | Digestive | ICD-9: 750, 751; ICD-10: Q38-Q40, Q41-Q45 |  |
|  | Other Urinary (genital anomalies) | ICD-9: 752; ICD-10:Q54 |  |
|  | Musculoskeletal | ICD-9: 754, 755, 756; ICD-10: Q65-Q68, Q743, Q763, Q7641, Q7642, Q75-Q79 |  |
|  | Respiratory | ICD-9: 748 (excluded 748.1); ICD-10: Q30-Q34 (excluded Q30.1, Q30.2, Q30.8) |  |
|  | Chromosomes | ICD-9: 758; ICD-10: Q554, Q90-Q99 |  |
|  | Skin | ICD-9: 757; ICD-10: N6482, Q80-Q84 |  |
|  | Others/Unspecified | ICD-9: 759; ICD-10: E7871, E7872, Q85, Q87, Q89, Q992 |  |
|  | **Maternal covariates** | **Code** | **Timing and Definition** |
|  | **Maternal health (8)** | | |
|  | Hypertension | ICD-9: 401-405 ICD-10: I10~I13,I15, N26.2 | Pre- and During-gestational period;  ≥1 ICD-9/10 codes in out- or inpatient setting |
|  | Hyperlipidemia | ICD-9: 272; ICD-10: E75.2, E75.3, E75.5, E75.6, E77, E78, E88.1, E88.2, E88.89 |  |
|  | Charlson Comorbidity Index [3] | **17 comorbid conditions:**  Acute myocardial infarction: ICD-9: 410,412,428; ICD-10: I21, I22, I252;  Congestive heart failure: ICD-9: 428; ICD-10: I50;  Peripheral vascular disease: ICD-9: 441, 4439, 7854, V434; ICD-10: I71, I790, I739, R02, Z958, Z959;  Cerebral vascular accident: ICD-9: 430–438; ICD-10: G45, G46, H340, I60, I61, I62, I63, I64, I65, I66, I67, I68, I69;  Dementia: ICD-9: 290; ICD-10: F00, F01, F02, F051;  Chronic pulmonary disease: ICD-9: 490–505; ICD-10: J40–J47, J60–J66;  Rheumatic disease: ICD-9: 7100, 7101, 7104, 7141,7142, 71481, 725; ICD-10: M05, M06, M32–M34, M351, M353;  Peptic ulcer disease: 531–534; ICD-10: K25–K28;  Mild liver disease/ moderate: ICd-9: 5712, 5714–5716; ICD-10: K702, K703, K717, K73, K74;  Moderate or severe liver disease: ICD-9: 5722, 5723, 5724, 5728; ICD-10: K729, K766, K767, K721;  Diabetes without complication: 2500–2503, 2507; ICD-10: E109, E119, E139, E149, E101, E111, E131, E141, E105, E115, E135, E145;  Diabetes with complication: 2504–2506; ICD-10: E102, E112, E132, E142 E103, E113, E133, E143 E104, E114, E134, E144;  Hemiplegia or paraplegia: ICD-9: 3441, 342; ICD-10: G81, G82;  N053, N054, N055, N056, N072, N073, N074, N18, N19, N25, Z490– Z492, Z940, Z992;  Any malignancy: ICD-9: 140–172, 174–1958, 200–208; ICD-10: C00–C26, C30–C34, C37– C41, C43, C45–C58, C60– C76, C80–C85, C88, C90–C96;  Metastatic solid tumor: ICD-9: 196–1991; ICD-10: C77–C80;  AIDS: 042–044; ICD-10: B20–B22, B24 |  |
|  | Kidney disease by ICD codes | ICD-9: 582, 583, 585, 586, 588, V420, V451, V56; ICD-10: N01, N03, N052, | Pre-existing maternal CKD: ≥1 inpatient ICD-9/10-CM code or ≥2 outpatient ICD-9/10-CM codes separated by ≥90 days and within 1 year, with first qualifying evidence before conception or within 280 days before the index pregnancy. Gestationally recognized CKD: the same diagnostic chronicity criteria with first qualifying evidence from conception to delivery; these cases may include previously unrecognized CKD rather than incident gestational CKD. |
|  | Kidney disease by eGFR | Taiwan modified MDRD equation for adults:  eGFR= 175 × SCr^-1.154^ ×age ^-0.203^ × 0.742 [if female]  Schwartz-bedside equation for children aged≥ 2 years:  eGFR= 0.413× (height in cm/SCr) | Maternal laboratory exposure: any eGFR <60 mL/min/1.73 m² during pregnancy was classified as reduced maternal kidney function, not definitive CKD unless supported by diagnostic chronicity. Offspring laboratory component: any bedside Schwartz eGFR <60 mL/min/1.73 m² at age ≥2 years was classified as reduced kidney function; a single value was not considered definitive CKD in isolation. |
|  | Gestational diabetes | ICD-9: 648.83; ICD10: O24 | During-gestational periods;  ≥1 ICD-9/10 codes in out- or inpatient setting |
|  | Gestational hypertension [4] | 1.pre-existing HTN: ICD-9: 642.0x, 642.1x, 642.2x; ICD-10: O10 2.gestational HTN: ICD-9: 642.3x, 642.9x; ICD-10: O13.1,O13.2, O13.3, O13.9; O16.1, O16.2, O16.3, O16.9 |  |
|  | (Pre)eclampsia [4] | ICD-9: 642.5x, 642.6x, 642.7x ; ICD-10:O14, O15, O11 |  |
|  | Anemia [5] | anemia during pregnancy (ICD-9: 648.23; ICD-10: O99.0,), excluding hemorrhage: O99.1 (coagulation defects: O46.0, O67.0, O72.3) |  |
|  | (Other) Gestational-related complications [6] (excluded gestational hypertension, hyperlipidemia, diabetes, preeclampsia and anemia) | ICD-9: 640, 641, 642, 643, 644, 645, 646,647,648,649 ICD-10: O100-O104, O109, O111-O113, O119-O122, O131-O133, O139-O142, O149-O152, O159, O161-O163, O169, O200, O208-O212, O218, O219, O230-O235, O239-O241, O243, O244, O248, O249, O251-O253, O260-O264, O266-O269, O290-O296, O298, O299, O310, O330, O440, O441, O450, O458, O459, O460, O468, O469, O470, O471, O479-O481, O600-O602, O670, O678, O679, O861, O862, O905, O906, O908, O980-O998, O9A1-O9A5 |  |
|  | **Medication use [7]** | **Anatomy of Therapeutic Classification code** | Pre-gestational period:  ≥1 prescription in outpatients  Gestational period;  ≥1 prescription in out- or in-patient setting |
|  | Anti-microbial agents | J01; Aminoglycosides (J01GB) |  |
|  | Systemic glucocorticoids [2] | betamethasone, dexamethasone, methylprednisolone, prednisolone, triamcinolone, and hydrocortisone; excluded topical use of preparations |  |
|  | NSAID or aspirin | M01AA, M01AB, M01AC, M01AE, M01AG, M01AH, M01AX (excld M01AX05)  B01AC06 (aspirin) |  |

References:

1. 2016 Annual Report on Kidney Disease in Taiwan. National Health Research Institutes, Hsinchu, Taiwan. Available at https://lib.nhri.edu.tw/NewWeb/nhri/ebook/39000000431630.pdf. Accessed at March 1, 2023.
2. Tain YL, Li LG, Kuo HC and Hsu CN. Gestational Exposure to Maternal Systemic Glucocorticoids and Childhood Risk of CKD. Am J Kidney Dis. 2024 Mar 11: S0272-6386(24)00669-3. doi: 10.1053/j.ajkd.2024.01.523.
3. Quan H, Sundararajan V, Halfon P, et al. Coding algorithms for defining comorbidities in ICD-9-CM and ICD-10 administrative data. Medical care 2005:1130-9
4. Fingar KR, Mabry-Hernandez I, Ngo-Metzger Q, et al. Delivery Hospitalizations Involving Preeclampsia and Eclampsia, 2005–2014: Statistical Brief #222. 2017 Apr. In: Healthcare Cost and Utilization Project (HCUP) Statistical Briefs [Internet]. Rockville (MD): Agency for Healthcare Research and Quality (US); 2006 Feb-. Table 7, ICD-9-CM diagnosis codes defining preeclampsia/eclampsia and other types of hypertension complicating pregnancy, childbirth, and the puerperium. Available from: https://www.ncbi.nlm.nih.gov/books/NBK442039/table/sb222.t7/ (assessed 10/10/2020)
5. Sharma AJ, Ford ND, Bulkley JE, et al. Use of the Electronic Health Record to Assess Prevalence of Anemia and Iron Deficiency in Pregnancy. *The Journal of Nutrition*, 2021; 151: 3588–3595.
6. Centers for Disease Control and Prevention. How Does CDC Identify Severe Maternal Morbidity? Available from https://www.cdc.gov/reproductivehealth/maternalinfanthealth/smm/severe-morbidity-ICD.htm. (assessed 10/10/2022)
7. Schreuder MF, Bueters RR, Huigen MC, et al. Effect of drugs on renal development. *Clinical Journal of the American Society of Nephrology.* 2011;6(1):212-217.

Supplementary Table 2. Offspring composite kidney outcomes by maternal kidney disease status and gestational timing in the maternal eGFR subcohort (n = 11,551)

| **Maternal exposure timing** | **Exposed children in maternal subcohort, n (%)** | | **Composite outcomes among exposed, n (%)** | | **Any CKD / reduced eGFR, n (%)** | | **CAKUT-related CKD, n (%)** | | **No-CAKUT-related CKD, n (%)** | | **Progressive kidney event, n (%)** | |  |
| --- | --- | --- | --- | --- | --- | --- | --- | --- | --- | --- | --- | --- | --- |
| Maternal kidney disease or reduced kidney function | 328 (2.84) | | 28 (8.54) | | 28 (8.54) | | 13 (3.96) | | 10 (3.05) | | 2 (0.61) | |  |
| CKD before pregnancy | 189 (1.64) | | 19 (10.05) | | 19 (10.05) | | 9 (4.76) | | 8 (4.23) | | 0 (0.00) | |  |
| CKD or reduced eGFR during pregnancy | 170 (1.47) | | 13 (7.65) | | 13 (7.65) | | 7 (4.12) | | 4 (2.35) | | 2 (1.18) | |  |
| 1st trimester (≤13 weeks) | | 40 (0.35) | | 4 (10.00) | | 4 (10.00) | | 2 (5.00) | | 2 (5.00) | | 0 (0.00) | |
| 2nd trimester (14-26 weeks) | | 49 (0.42) | | 8 (16.33) | | 8 (16.33) | | 5 (10.20) | | 3 (6.12) | | 1 (2.04) | |
| 3rd trimester (≥27 weeks) | | 119 (1.03) | | 9 (7.56) | | 9 (7.56) | | 5 (4.20) | | 3 (2.52) | | 2 (1.68) | |

CKD, chronic kidney disease; eGFR, estimated glomerular filtration rate; CAKUT, congenital anomalies of the kidney and urinary tract.

Percentages in the exposure column use the full maternal eGFR subcohort (n=11,551) as the denominator; percentages in outcome columns use the exposed children in the same row as the denominator.

1. Maternal kidney disease in the primary cohort was based on ICD-defined CKD meeting diagnostic chronicity criteria. In the maternal eGFR subcohort, reduced maternal kidney function was additionally defined as eGFR <60 ml/min/1.73 m² using the Taiwan-modified MDRD equation.

2. CKD before the index pregnancy was defined as qualifying maternal CKD evidence before conception or within 280 days before conception. CKD or reduced kidney function during pregnancy was defined from conception to delivery and summarized by gestational timing.

3. Timing categories were not mutually exclusive because mothers could have qualified evidence in more than one time window; therefore, timing-specific rows should not be summed.

4. The composite kidney outcome: ICD-defined CKD, including CAKUT and non-CAKUT CKD, reduced eGFR (<60 ml/min/1.73m2), and progressive kidney event, including kidney replacement therapy initiation, eGFR declined >=30% from baseline

Supplementary Table 3 Maternal characteristics by offspring composite kidney outcome status in the maternal eGFR subcohort (n = 11,551)

| **Maternal covariates** | | | | All children  (n=11551) | Composite outcome cases (n=729) | |  | No composite outcome controls  (n=10822) | | *p-value* |
| --- | --- | --- | --- | --- | --- | --- | --- | --- | --- | --- |
|  |  |  |  |  |  |  |  |  |  |  |
|  | **Maternal age (year), n (%)** | | |  |  |  |  |  |  | 0.1782 |
|  |  | <35 | | 8176 | 532 | (72.98) |  | 7644 | (70.63) |  |
|  |  | ≧35 | | 3375 | 197 | (27.02) |  | 3178 | (29.37) | . |
| **Gestational medication use, n (%)** | | | |  |  |  |  |  |  |  |
|  | NSAIDs/aspirin | | | 2186 | 149 | (20.44) |  | 2037 | (18.82) | 0.2809 |
|  | Glucocorticoids (GC) | | | 1882 | 157 | (21.54) |  | 1725 | (15.94) | 0.0001 |
|  | Anti-microbial | | | 6540 | 439 | (60.22) |  | 6101 | (56.38) | 0.0427 |
|  |  | Aminoglycosides | | 55 | 2 | (0.27) |  | 53 | (0.49) | 0.4135 |
| **Prior medication use, n (%)** | | |  |  |  |  |  |  |  |  |
|  | NSAID/aspirin | | | 7008 | 445 | (61.04) |  | 6563 | (60.64) | 0.8316 |
|  | Glucocorticoids (GC) | | | 2666 | 171 | (23.46) |  | 2495 | (23.05) | 0.8031 |
|  | Anti-microbial | | | 7304 | 474 | (65.02) |  | 6830 | (63.11) | 0.301 |
|  |  | Aminoglycosides | | 1352 | 89 | (12.21) |  | 1263 | (11.67) | 0.6619 |
| **Gestational comorbid conditions, n (%)** | | | | |  |  |  |  |  |  |
|  | Gestational diabetes | | | 373 | 19 | (2.61) |  | 354 | (3.27) | 0.3257 |
|  | Gestational hypertension | | | 384 | 28 | (3.84) |  | 356 | (3.29) | 0.4216 |
|  | Pre-eclampsia | | | 187 | 8 | (1.1) |  | 179 | (1.65) | 0.2490 |
|  | Anemia | | | 84 | 2 | (0.27) |  | 82 | (0.76) | 0.1371 |
|  | Other gestational complications | | | 6325 | 407 | (55.83) |  | 5918 | (54.68) | 0.5477 |
| **Prior health condition, n (%)** | | |  |  |  |  |  |  |  |  |
|  | Congestive heart failure | | | 63 | 5 | (0.69) |  | 58 | (0.54) | 0.5947 |
|  | Peripheral vascular diseases | | | 34 | 3 | (0.41) |  | 31 | (0.29) | 0.5463 |
|  | Cerebral vascular accident | | | 103 | 5 | (0.69) |  | 98 | (0.91) | 0.5414 |
|  | Pulmonary disease | | | 848 | 60 | (8.23) |  | 788 | (7.28) | 0.3417 |
|  | Connective tissue disorder | | | 263 | 32 | (4.39) |  | 231 | (2.13) | 0.0001 |
|  | Peptic ulcer | | | 1546 | 100 | (13.72) |  | 1446 | (13.36) | 0.7848 |
|  | Liver diseases | | | 1060 | 66 | (9.05) |  | 994 | (9.18) | 0.9052 |
|  | Diabetes | | | 489 | 33 | (4.53) |  | 456 | (4.21) | 0.6844 |
|  | Diabetes complications | | | 83 | 5 | (0.69) |  | 78 | (0.72) | 0.9140 |
|  | Cancer | | | 175 | 15 | (2.06) |  | 160 | (1.48) | 0.2153 |
|  | metastatic solid tumor | | | 22 | 1 | (0.14) |  | 21 | (0.19) | 0.7332 |
|  | Hypertension | | | 486 | 36 | (4.94) |  | 450 | (4.16) | 0.3099 |
|  | Hyperlipidemia | | | 465 | 29 | (3.98) |  | 436 | (4.03) | 0.9462 |
| **Maternal CKD any time** | | | | 328 | 28 | (3.84) |  | 300 | (2.77) | 0.0927 |
|  | Before pregnancy (within 280 days before conception), n (%) | | | 189 | 19 | (2.61) |  | 170 | (1.57) | 0.0329 |
|  | During pregnancy, n (%) | | | 170 | 13 | (1.78) |  | 157 | (1.45) | 0.4705 |
|  |  | 1st trimester (≤13 week) | | 40 | 4 | (0.55) |  | 36 | (0.33) | 0.3365 |
|  |  | 2nd trimester (14-26 week) | | 49 | 8 | (1.1) |  | 41 | (0.38) | 0.0039 |
|  |  | 3rd trimester (≥27 weeks) | | 119 | 9 | (1.23) |  | 110 | (1.02) | 0.5724 |
| **Available maternal eGFR during pregnancy** | | | | 10070 | 679 |  |  | 9391 |  |  |
| **Maternal eGFR group, n (%)** | | | | |  |  |  |  |  | 0.0001 |
|  | ≥90 | | | 8988 | 601 | (88.51) |  | 8387 | (89.31) |  |
|  | 60-<90 | | | 820 | 44 | (6.48) |  | 776 | (8.26) |  |
|  | 45 | | | 122 | 13 | (1.91) |  | 109 | (1.16) |  |
|  | <45 | | | 140 | 21 | (3.09) |  | 119 | (1.24) |  |
| **1st trimester, n (%)** | | | |  |  |  |  |  |  | 0.1803 |
|  | ≥90 | | | 2333 | 148 | (87.57) |  | 2185 | (87.12) |  |
|  | 60-<90 | | | 285 | 15 | (8.88) |  | 270 | (10.77) |  |
|  | 45-<60 | | | 26 | 1 | (0.59) |  | 25 | (1.00) |  |
|  | <45 | | | 33 | 5 | (2.96) |  | 28 | (1.12) |  |
| **2nd trimester, n (%)** | | | |  |  |  |  |  |  | 0.0667 |
|  | ≥90 | | | 1997 | 143 | (92.26) |  | 1854 | (93.31) |  |
|  | 60-<90 | | | 94 | 4 | (2.58) |  | 90 | (4.53) |  |
|  | 45-<60 | | | 19 | 3 | (1.94) |  | 16 | (0.81) |  |
|  | <45 | | | 32 | 5 | (3.23) |  | 27 | (1.36) |  |
| **3rd trimester, n (%)** | | | |  |  |  |  |  |  | 0.0152 |
|  | ≥90 | | | 4658 | 310 | (87.32) |  | 4348 | (88.81) |  |
|  | 60-<90 | | | 441 | 25 | (7.04) |  | 416 | (8.50) |  |
|  | 45-<60 | | | 77 | 9 | (2.54) |  | 68 | (1.39) |  |
|  | <45 | | | 75 | 11 | (3.10) |  | 64 | (1.31) |  |

CKD, chronic kidney disease; eGFR, estimated glomerular filtration rate; CAKUT, congenital anomalies of the kidney and urinary tract.

1. The composite kidney outcome in offspring: ICD-defined CKD, including CAKUT and non-CAKUT CKD, reduced eGFR (<60 ml/min/1.73m2), and progressive kidney event, including kidney replacement therapy initiation, eGFR declined >=30% from baseline
2. Maternal kidney disease was defined as maternal CKD identified before offspring birth by ICD-9/10 diagnosis codes or maternal eGFR <60 ml/min/1.73 m². CKD during pregnancy was defined as CKD identified from conception to delivery and was classified by gestational timing: first trimester, ≤13 weeks; second trimester, 14–26 weeks; and third trimester, ≥27 weeks.
3. Maternal eGFR was calculated using the Taiwan-modified MDRD equation: 175 × SCr ^-1.154^ × Age ^-0.203^ × (0.742 [female])
4. CKD timing categories were not mutually exclusive because mothers could have qualified CKD evidence in more than one time window; therefore, timing-specific rows should not be summed.

Supplementary Figure 1. Conceptual directed acyclic graph for the association between maternal kidney disease and observed offspring composite kidney outcomes.

Solid boxes and arrows indicate measured variables and modeled pathways; dashed boxes and arrows indicate conceptual unmeasured variables or pathways. The dashed exposure–outcome arrow represents pathways not captured by measured pregnancy and perinatal factors.

Measured baseline maternal factors were adjusted for in regression models. Pregnancy and perinatal factors were considered potential mediators or conditional adjustment variables. Familial/shared factors represent unmeasured common-cause pathways, whereas healthcare contact and testing represent an ascertainment pathway affecting detection of maternal kidney dysfunction and offspring kidney outcomes.
